# Leemoo, a Dietary Assessment and Nutritional Planning Software, Using Fuzzy Logic

**DOI:** 10.5812/ijem.10169

**Published:** 2013-10-11

**Authors:** Hanieh-Sadat Ejtahed, Mohammad Mahdi Sarsharzadeh, Parvin Mirmiran, Golaleh Asghari, Emad Yuzbashian, Fereidoun Azizi

**Affiliations:** 1 Nutrition and Endocrine Research Center, Obesity Research Center, Research Institute for Endocrine Sciences, Shahid Beheshti University of Medical Sciences, Tehran, IR Iran; 2 Department of Clinical Nutrition and Dietetics, Faculty of Nutrition Sciences and Food Technology, National Nutrition and Food Technology Research Institute, Shahid Beheshti University of Medical Sciences, Tehran, IR Iran; 3 Endocrine Research Center, Research Institute for Endocrine Sciences, Shahid Beheshti University of Medical Sciences, Tehran, IR Iran

**Keywords:** Nutrition Software, Leemoo, Fuzzy Logic, Food Groups

## Abstract

**Background:**

Leemoo is a user-friendly software designed for nutritional planning and dietary assessment applications by cooperation of Research Institute for Endocrine Sciences of Shahid Beheshti University of Medical Sciences, which uses fuzzy logic for the first time. It provides a range of recommended servings for food groups, which are easier for people to follow.

**Objectives:**

The aim of this research was to describe the applications of this newly introduced software.

**Methods and Materials:**

Leemoo contains the following databases: food, nutrients, MyPyramid equivalents and physical activity. These databases facilitate diet analysis and planning. The food composition database includes 2920 Iranian food items, with the nutrient composition for up to 64 nutrients.

**Results:**

All databases are flexible for updating. The program can calculate the amounts of nutrients and food group intakes and compare these with Dietary Reference Intakes and MyPyramid recommendations. This software has the ability of nutritional planning and estimates normal intake ranges of seven food groups based on person need.

**Conclusions:**

This software was envisaged for use by health professionals, researchers and ordinary people and can be recommended for educational purpose and nutrition research in Iran. Future studies must be conducted to evaluate the effects of this software on users’ dietary habits and nutrition knowledge.

## 1. Background

Improving the dietary habits of people is an important task, which helps to decrease the morbidity and mortality of many chronic diseases and to fight the obesity problem. Education is one of the methods to encourage healthy eating. Mass media, including print media, television, the Internet and software are the educational tools required (1, 2). Using computers for dietary assessment has cognitive advantages. These include enhanced communication through pictures, decreased bias, increased flexibility, and collecting data in a neutral environment (3, 4). Computerized assessment can provide automatic feedback, tailored for the individual which has motivating and informative effect. Feedback may provide the form of graphs or tables representing the adequacy of nutritional intake, health risks associated with low or high intakes, and related nutrition recommendations. This information can improve health condition (5, 6). 

Assessing the current dietary intake and planning nutritious menus for individuals and groups is a complex task that researchers have tried to computerize since the early 1960s (7), and a number of software had been developed during recent years such as ‘NutPlan, Nutri-Educ and Iranian Food Consumption Program (IFCP)’ (8-10). Nutri-Educ helps the users to build well-balanced meals and analyzes the composition of meals (8) and IFCP is a nutrient calculation system of Iranian dishes (10).

If nutritional education is necessary to optimize the diet, it is important that changes in the dietary habit not be drastic. Therefore, providing an optimal intake “range” rather than a single optimal point of intake is more applicable for people (11). In the MyPyramid guidance system, the recommended amounts of food groups are absolute values. Therefore, it is hard to follow the MyPyramid exactly every day. For solving this limitation, we introduce Fuzzy guideline that moderate energy and nutrient by the iterative algorithm (12).

Persian dietary assessment and planning software for Iranians does not exist. Therefore, we have developed a software for dietary planning and analysis. Leemoo is a user-friendly, self-administered tool that can be used by both nutrition professionals and ordinary people to evaluate the adequacy of diets. The current dietary software was designed to monitor and assess meals and food items consumed by individuals according to dietary databases including MyPyramid equivalents and nutrients. Moreover, this software creates an association between nutrition and fuzzy sets, which can define the desirability for a recommended amount of each food group, providing a range of recommended servings of food groups. This software derived the fuzzy pyramid from 1600 kilocalories to 4000 kilocalories by distance of 200 kilocalories (12).

## 2. Objectives

In the current study, we tried to describe the applications of this newly introduced software to inform potential users of its existence.

## 3. Materials and Methods

### 3.1. Design

The software has been developed by cooperation of the Research Institute for Endocrine Sciences, of the Shahid Beheshti University of Medical Sciences. Leemoo is a Persian software and presently available in Iran.

### 3.2. Leemoo Structure

#### 3.2.1. Database Features

The database of this tool consists of a food database, a nutrients database, the MyPyramid equivalents database and a physical activity database. All databases in this software are a backbone of the system and flexible for updating the data. The food composition database includes 2920 Iranian food items, with the nutrient composition of these foods for up to 64 nutrients. Every food item has basic components such as energy, fatty acids, macro- and micronutrients and includes other descriptors such as food name, food group, subgroup, photograph, and unit. Food amounts are provided in gram weights and in common household measures. The source of the food database was the United States Department of Agriculture (USDA), a freely accessible database, which contains the composition of all raw and processed foods and includes both single food items such as potato and pineapple and also dishes such as pizza (13, 14). The USDA has conducted periodic nationwide surveys on the kinds and amounts of foods consumed by people, using the 24-h dietary recall method to update its food database (15). Common Iranian homemade foods such as “ghormesabzi” have been added in this software. Moreover, it has the facility that users can add their food recipes to the database. 

The Dietary Reference Intakes (DRIs) are used for estimation of nutrient requirements based on sex and age groups (16). USDA’s MyPyramid food guidance system is used to conduct food group analysis and dietary planning (17). Energy costs of different physical activities such as different kinds of home activities, occupation, sports and walking are considered in the physical activity database (18). There is a food subgroup database for analyzing of the weekly diet and averaging multiple days of food subgroups’ intake.

#### 3.2.2. Software Features 

This software has two main applications, including dietary assessment and nutritional planning. At first, information, including personal characteristics such as name, age, sex, weight, height and physical activity are collected through specifically designed self-guided questionnaires.

## 4. Results

**Figure 1.**
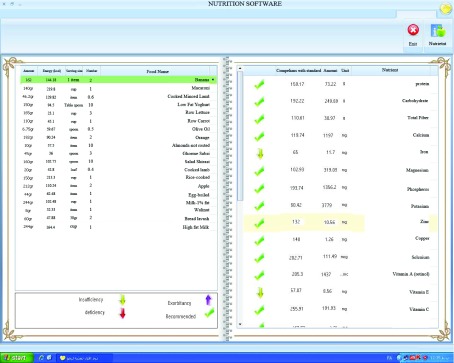
Leemoo’s Dietary Assessment Examples

Leemoo software can calculate body mass index (BMI) and estimate the amount of energy needed for any person by estimated energy requirement (EER) equations ( 19 ). Users can enter their weights every day and check the trend of their weight and BMI changes over time. To assess the dietary status, users should enter the main data collected on food consumed. The methods for food data collection include 24-hour dietary recalls and food records. The food name is simply entered with no need for entry of codes. Then, the amount of consumed foods should be provided in gram weights or common household measures. The software can calculate the amounts of nutrients and food group intakes and compare them with DRIs and MyPyramid recommendations (Figure 1). Moreover, this program can give visual alarm signals to people about any problems in their diet, and it has educational messages for users as well. 

**Figure 2.**
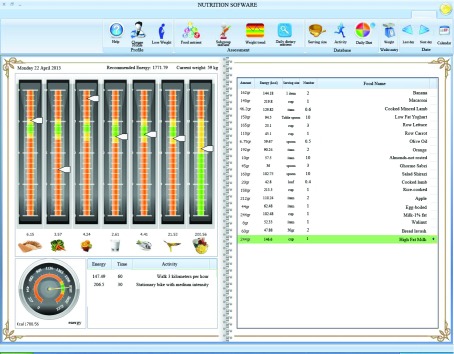
Examples of Leemoo’s Recommended Servings of Food Groups

For nutritional planning, the software can calculate the energy need and estimate the recommended servings of seven food groups, including fruits, vegetables, grains, meats, milk, oils and fat and added sugar. This software uses the fuzzy pyramid pattern. Therefore, there is a normal intake range rather than a single optimal point of intake. This program provides the desirability for the amount of intake for food groups in three particular ranges: 1- Normal range (green color), 2- Attention range (yellow color), 3- Danger range (red color) except for fat and added sugar groups. For added sugar and fat, we should have moderation, which is in contrast to other food groups that we should have adequacy. So their recommendations in fuzzy pattern are different from other food groups (Figure 2). 

## 5. Discussion

Leemoo has been developed to help make wise and healthy food choices. This software has potential to become an important tool for nutritional assessment, evaluation of the dietary adequacy of meals or diets and promotion of healthy eating in Iran. Moreover, it can be considered as an educational tool in nutrition. However, intervention and evaluation studies must be conducted to ascertain the extent which using Leemoo improves the users’ knowledge and modifies their dietary habits. If it investigated and confirmed, the Leemoo would be an ideal nutritional software to help fight the obesity problem. Moreover, if these studies prove successful, the use of this software could be valuable in clarifying diet-disease associations in epidemiologic studies. 

One of this software’s unique features is the fuzzy logic application, which makes this pattern more applicable for people. This is the first nutritional planning software developed, using fuzzy pyramid recommendation. Fuzzy logic is a mathematical approach to deal with systems that cannot be defined precisely. Nutrient and food group requirements fall into this category. Our program creates an association between nutrition and fuzzy sets. This software gives an optimal intake range for each of seven food groups that are so easier for people to follow and enhance the dietary adherence (12). Furthermore, it considers the amount of energy needed for any person and gives recommendations based on the EER. Another important feature is its being user-friendly because of the ease of data entry, ability to edit the food lists, and optional expression of food portion. It has advice components which have instructive and motivating effects. It has meal-based questions which have been shown to result in more accurate reporting than questions regarding individual foods (3). Observation of the photographs of foods and their portion sizes can prompt the respondent’s memory and improve the accuracy of reporting actual consumption. 

Iranian Food Consumption Program is a nutrient calculation system which only calculates nutrients of Iranian dishes through an atlas of photographs and investigates the possible associations between dietary habits and cardiovascular disease risk factors. It does not have dietary planning application. Leemoo has the ability of dietary assessment and nutritional planning and estimates normal intake ranges of food groups. The language of IFCP is both Persian and English. In this program, homemade foods are not considered as individual items and users must enter recipe ingredients (10). However, Common Iranian homemade foods have been added in Leemoo, which are easier for ordinary people to use. Nutri-Educ is French nutrition software, which uses fuzzy arithmetic and heuristic search algorithms too. Its main goal is to help people to assess their diets and balance their meals. It transforms the initial meal into a well balanced one based on users' energy needs and their medical problems related to nutrition (9). Our software does not include specific considerations for various diseases. NutPlan is multi-lingual dietary software for Eastern European and West Balkan countries. It has multiple functions including individual and group nutrition planning, creating food labels and nutrient intake assessment. It can be used in the food industry and can be recommended for educational purposes (8). It has more applications compared with Leemoo. However, it does not use fuzzy logic. 

This software has a few limitations. It has been designed for healthy people, and it does not have specific considerations for various diseases. For example, it does not have the facility of “carbohydrate counting” for diabetic patients. Adding brand names of food items and specific considerations for chronic diseases may optimize its utility. The initial version of the software is in the process of being completed in future versions. This software was not examined for confirmation of its validity in clinical settings. For the future, we intend to make medical evaluation studies to evaluate the effects of this software and fuzzy dietary pattern on users’ dietary patterns.

The first version of the software has been finalized, and it is flexible for editing database. Leemoo is the first nutritional software which uses fuzzy logic in Iran and has two main applications: dietary assessment and nutritional planning. It provides a range of recommended servings for food groups which are easier for people to follow. This software needs to be examined for confirmation of its validity in future studies.

## References

[1] Contento Isobel (2007). Nutrition education: linking research, theory, and practice..

[2] Pieniak Z, Perez-Cueto F, Verbeke W (2009). Association of overweight and obesity with interest in healthy eating, subjective health and perceived risk of chronic diseases in three European countries.. Appetite..

[3] Bakker I, Twisk JW, van Mechelen W, Mensink GB, Kemper HC (2003). Computerization of a dietary history interview in a running cohort; evaluation within the Amsterdam Growth and Health Longitudinal Study.. Eur J Clin Nutr..

[4] Kohlmeier L, Mendez M, McDuffie J, Miller M (1997). Computer-assisted self-interviewing: a multimedia approach to dietary assessment.. Am J Clin Nutr..

[5] Brug J (1999). Dutch research into the development and impact of computer-tailored nutrition education.. Eur J Clin Nutr..

[6] Probst Yasmine C, Tapsell Linda C (2005). Overview of Computerized Dietary Assessment Programs for Research and Practice in Nutrition Education.. J Nutr Edu Behav..

[7] Eckstein EF (1967). Menu planning by computer: the random approach.. J Am Diet Assoc..

[8] Buisson JC (2008). Nutri-Educ, a nutrition software application for balancing meals, using fuzzy arithmetic and heuristic search algorithms.. Artif Intell Med..

[9] Gurinovic M, Kadvan A, Bucchini L, Matthys C, Torres D, Novakovic R (2010). EURRECA nutritional planning and dietary assessment software tool: NutPlan.. Eur J Clin Nutr..

[10] Rafiei M, Boshtam M, Marandi A, Jalali A, Vakili R (2002). The Iranian food consumption program (IFCP), a unique nutritional software in Iran.. Iranian J Publ Health..

[11] Wirsam B, Uthus EO (1996). The use of fuzzy logic in nutrition.. J Nutr..

[12] Asghari G, Ejtahed H, Sarsharzadeh M, Nazeri P, Mirmiran P (2013). Designing Fuzzy Algorithms to Develop Healthy Dietary Pattern.. Int J Endocrinol Metab..

[13] (2008). USDA Food and Nutrient Database for Dietary Studies. Agricultural Research Service, Food Surveys Research Group..

[14] U.S. Department of Agriculture Agricultural Research Service. (2011). Nutrient Data Laboratory Home Page..

[15] Raper Nancy, Perloff Betty, Ingwersen Linda, Steinfeldt Lois, Anand Jaswinder (2004). An overview of USDA's Dietary Intake Data System.. J Food Comp Analysis..

[16] Trumbo Paula, Yates Allison A, Schlicker Sandra, Poos Mary (2001). Dietary reference intakes: vitamin A, vitamin K, arsenic, boron, chromium, copper, iodine, iron, manganese, molybdenum, nickel, silicon, vanadium, and zinc.. J Am Diet Assoc..

[17] U.S. Department of Agriculture and U.S. (2006.). Department of Health and Human Services..

[18] Montoye Henry J (2000). Energy costs of exercise and sport..

[19] Krause Marie V, Mahan L Kathleen, Escott-Stump Sylvia, Raymond Janice L (2012). Krause's food & the nutrition care process..

